# Aerobic Glycolysis Is Required for Spatial Memory Acquisition But Not Memory Retrieval in Mice

**DOI:** 10.1523/ENEURO.0389-18.2019

**Published:** 2019-02-22

**Authors:** Richard A. Harris, Asad Lone, Heeseung Lim, Francisco Martinez, Ariel K. Frame, Timothy J. Scholl, Robert C. Cumming

**Affiliations:** 1 Department of Biology, University of Western Ontario, London, Ontario N6A 5B7, Canada; 2 Department of Medical Biophysics, University of Western Ontario, London, Ontario N6A 5C1, Canada; 3 Robarts Research Institute, University of Western Ontario, London, Ontario N6A 3K7, Canada; 4 Ontario Institute for Cancer Research, Toronto, Ontario M5G 0A3, Canada

**Keywords:** aerobic glycolysis, lactate, magnetic resonance spectroscopy, memory, metabolism, synaptic plasticity

## Abstract

The consolidation of newly formed memories and their retrieval are energetically demanding processes. Aerobic glycolysis (AG), also known as the Warburg effect, consists of the production of lactate from glucose in the presence of oxygen. The astrocyte neuron lactate shuttle hypothesis posits that astrocytes process glucose by AG to generate lactate, which is used as a fuel source within neurons to maintain synaptic activity. Studies in mice have demonstrated that lactate transport between astrocytes and neurons is required for long-term memory formation, yet the role of lactate production in memory acquisition and retrieval has not previously been explored. Here, we examined the effect of dichloroacetate (DCA), a chemical inhibitor of lactate production, on spatial learning and memory in mice using the Morris water maze (MWM). *In vivo* hyperpolarized ^13^C-pyruvate magnetic resonance spectroscopy revealed decreased conversion of pyruvate to lactate in the mouse brain following DCA administration, concomitant with a reduction in the phosphorylation of pyruvate dehydrogenase. DCA exposure before each training session in the MWM impaired learning, which subsequently resulted in impaired memory during the probe trial. In contrast, mice that underwent training without DCA exposure, but received a single DCA injection before the probe trial exhibited normal memory. Our findings indicate that AG plays a key role during memory acquisition but is less important for the retrieval of established memories. Thus, the activation of AG may be important for learning-dependent synaptic plasticity rather than the activation of signaling cascades required for memory retrieval.

## Significance Statement

Neuronal activation is an energetically demanding process. The brain is mainly fueled by glucose, yet a substantial portion of this metabolite is converted to lactate despite the presence of adequate oxygen, a phenomenon known as aerobic glycolysis (AG). The transport of lactate between astrocytes and neurons is key for learning and memory, yet the role of lactate production in these processes is poorly understood. Here we report that the administration of dichloroacetate (DCA), a chemical inhibitor of AG, attenuates the conversion of pyruvate to lactate in the brains of mice. DCA exposure impaired spatial learning but had no effect on the retrieval of an established memory. These observations suggest that lactate production may be required for memory acquisition but not retrieval.

## Introduction

Glucose is the primary fuel source within the human brain (Mergenthaler et al., 2013) and can be processed by various metabolic pathways to produce energy. Under normal oxygen tension, glucose is typically broken down in the cytosol to pyruvate, which then enters the mitochondria and undergoes oxidative decarboxylation by pyruvate dehydrogenase (PDH) to produce acetyl-CoA. Acetyl-CoA provides carbon atoms for the tricarboxylic acid cycle leading to the production of NADH and FADH_2_, ultimately generating 32–36 ATP molecules by oxidative phosphorylation. Under hypoxic conditions, pyruvate is preferentially converted to lactate by lactate dehydrogenase (LDHA) to produce two ATP molecules in a process known as anaerobic glycolysis. However, a third way to process glucose, termed aerobic glycolysis (AG), occurs when glucose is processed to generate lactate despite normal oxygen tension. Under resting conditions, AG accounts for 10*–*15% of glucose consumed in the human brain and increases to up to 40% following neuronal activation (Raichle et al., 1970; Boyle et al., 1994; Madsen et al., 1998, 1999; Powers et al., 2007). Recent evidence has implicated lactate, the end product of AG, in the regulation of synaptic plasticity and gene expression changes associated with learning and memory (Suzuki et al., 2011; Goyal et al., 2014; Yang et al., 2014; Shannon et al., 2016). Astrocytes are believed to be the primary cell type in the CNS that uses AG to generate lactate, which is subsequently transported to neurons to meet their high energy needs (Magistretti and Allaman, 2018). Astrocytic glycogen can also be mobilized via glycogenolysis to generate glucose for subsequent processing by AG to generate lactate (Brown et al., 2004). Numerous studies in rodents have shown that the inhibition of astrocytic glycogenolysis or lactate transport results in compromised learning and memory (Magistretti and Allaman, 2018). However, the effect of AG inhibition on cognitive processes has yet to be examined.

Dichloroacetate (DCA) is a blood*–*brain barrier (BBB)-permeable chemical that selectively inhibits PDH kinase (PDK; Kato et al., 2008). Phosphorylation of PDH by PDK results in a strong suppression of PDH activity, thereby favoring AG and lactate production (Kato et al., 2008). Based on its ability to inhibit PDK and promote optimal mitochondrial PDH activity, DCA has been used clinically to lower lactate levels in the blood and CSF of patients with congenital lactic acidosis (Stacpoole et al., 2003). In this study, we examined the effect of DCA-mediated inhibition of AG on spatial learning and memory in mice. Exposure to DCA readily prevented the phosphorylation of PDH and reduced the conversion of pyruvate to lactate within the brain. Moreover, DCA administration significantly impaired spatial learning but had no effect on the recall of established memories, suggesting that AG may play a role in memory acquisition but not retrieval.

## Materials and Methods

### Animals

Male C57BL/6J mice (RRID:IMSR_JAX:000664) were housed in groups under a 12 h light/dark cycle with ad libitum access to base chow (2018 Teklad Global Diet, Envigo) and tested at 9 months of age. All animal procedures were performed in compliance with the Canadian Council on Animal Care guidelines under an animal protocol approved by the University of Western Ontario animal care committee.

### Hyperpolarized 13C-pyruvate magnetic resonance spectroscopy

Images were acquired using a multinuclear-capable 3.0 Tesla MRI system (Discovery MR750 3.0 T; GE Healthcare). A custom-built dual-tuned ^1^H-^13^C solenoid radio frequency coil was used to facilitate inherent registration of ^1^H and ^13^C images. A buffered hyperpolarized [1-^13^C]pyruvate (95% enriched ^13^C content; Sigma-Aldrich) solution was produced using dynamic nuclear polarization (HyperSense, Oxford Instruments). The hyperpolarized solution had a final concentration of 150 mm pyruvate with pH 7.4 at 37°C and ∼10% polarization producing a signal enhancement factor of >10,000 for magnetic resonance spectroscopy imaging (MRSI). The *in vivo* spin-lattice relaxation time was ∼45 s. The imaging session consisted of fast imaging using steady-state free-precession ^1^H image acquisition (FIESTA) and hyperpolarized ^13^C MRSI. Before ^13^C MRSI, FIESTA images were acquired with the following imaging parameters: 30 × 30 mm field of view (FOV), 0.2 mm isotropic in-plane resolution, 0.4 mm slice thickness, repetition time (TR) = 10.3 ms, echo time = 5.2 ms, bandwidth = 12.58 Hz, and phase cycling = 8. For hyperpolarized ^13^C MRSI, a ∼0.3 ml bolus of the hyperpolarized [1-^13^C] pyruvate-buffered solution was injected over 10 s via a tail vein catheter and allowed to circulate for 15 s for cell uptake and conversion of the ^13^C-labeled pyruvate before imaging. The 2D MRSI was performed using a free induction decay chemical shift pulse sequence with the following parameters: 30 × 30 mm FOV, 2.5 mm isotropic in-plane resolution, slice thickness = 10∼15 mm, TR = 80 ms, spectral width = 5000 Hz, and number of points = 256. Total MRSI acquisition time was ∼12 s. DCA (Sigma-Aldrich) was freshly prepared at a concentration of 40 mg/ml in sterile saline and neutralized to pH 7.4 ± 0.1 using NaOH. Mice were given 30 min to recover following hyperpolarized ^13^C-pyruvate injection before injection of DCA at 200 mg/kg via tail vein catheter. Mice were then given an additional 30 min to recover, followed by another bolus of hyperpolarized ^13^C-pyruvate via a tail vein catheter. Mice immediately underwent a second ^13^C MRSI session, as previously described. Intravenous injection of DCA, as opposed to intraperitoneal injection used in learning and memory testing (described below), was necessary due to the technical requirement of maintaining exact head position during all stages of MRSI.

### Western blot analysis of brain extracts

Mice were sedated in a CO_2_ chamber and then immediately perfused with Dulbecco’s PBS, pH 7.4 containing 2 mm leupeptin, 0.1 mm pepstatin A, 100 mm EDTA, 1 mm PMSF, and 0.5 mm sodium orthovanadate. The brain was removed, and the frontal cortex of the right hemisphere was homogenized in an extraction buffer containing 50 mm Tris pH 7.5, 2% SDS, and protease and phosphatase inhibitors. Protein extracts were resolved by 10% SDS-PAGE, and electroblotted onto a PVDF membrane (Bio-Rad). Membranes were probed with the following antibodies: PDK1 (catalog #KAP-PK112D, Enzo Life Sciences; RRID:AB_1193509), PDH-E1α (pSer^232^; catalog #AP1063, Millipore; RRID:AB_10616070), PDH-E1α (catalog #ab110330, Abcam; RRID:AB_10858459), and β-actin (catalog #3700, Cell Signaling Technology; RRID:AB_2242334). Bands were detected using Luminata Forte chemiluminescence substrate (EMD Millipore) and imaged using a Chemidoc XRS System (Bio-Rad). Densitometric analysis was performed using Quantity One 1-D Analysis Software (Bio-Rad; RRID:SCR_014280).

### Water maze apparatus

The Morris water maze (MWM) consisted of a uniformly white circular pool with a diameter of 48 inches and a height of 30 inches (San Diego Instruments) and was divided into four fictive quadrants. The pool was filled with water and maintained at a temperature of 24°C using a 300 W submersible aquarium heater (Aqueon). Spatial visual cues in the form of different-shaped cardboard pictures were placed on the walls surrounding the water tank. Mice were first submitted to a single habituation session of three trials, in which the mouse was placed on circular plastic platform (diameter, 10.16 cm) positioned 1 cm below the surface of the water in the center of the pool for 15 s. Following habituation, mice were trained for 4 consecutive days, with four trials per day to find the location of the circular platform located in the center of the target quadrant. The release point for the first trial of the day corresponded to the cardinal directions (i.e., North, East, South, West), rotating clockwise with each new training day. For each subsequent trial session on a given day, mice were released into the water at a position that was a one-quarter turn clockwise from the previous trial release point. A trial lasted either until the mouse found the platform or 90 s had elapsed, at which point the mouse was gently guided to the platform and left there for 15 s before it was removed. Following each trial, mice were toweled dry and placed in their home cage for an intertrial interval of 10 min to allow recovery and ensure the consistency of timing between trials. On the fifth day, the platform was removed, and the mouse was released in the center of the tank and given 60 s to attempt to find the missing platform. A second probe trial was performed 1 week later, using the same procedure as the first probe trial. A flag trial was also performed in which the platform was placed back into the water within the quadrant that was opposite to that used during the training trials, and a small red plastic pole was inserted into the platform so as to create a visual cue. The mouse was released in the center of the tank and given 90 s to find the location of the platform. Visual data were collected and analyzed using ANY-maze version 4.98 (ANY-maze; RRID:SCR_014289). Animals were injected intraperitoneally with either saline or DCA (Sigma-Aldrich) at a dose of 200 mg/kg. One cohort of mice was injected with either saline or DCA 30 min before the start of each training session, while a second cohort of mice underwent normal training but received either a saline or DCA injection 30 min before the probe trial.

### Statistical analysis

A Welch’s paired *t* test was used to analyze the difference between the ratio of lactate to pyruvate for before and after DCA injection. A Welch’s unpaired *t* test was used to analyze band densitometry and memory performance for saline- and DCA-injected mice. A two-way ANOVA with repeated measures was used to analyze the differences among latency to the platform, path length, and mean speed for saline- and DCA-injected mice during training. Statistical evaluation was performed using RStudio version 0.97.551 (RStudio; RRID:SCR_000432).

## Results

### DCA reduces conversion of pyruvate to lactate in the mouse brain

**Figure 1.**
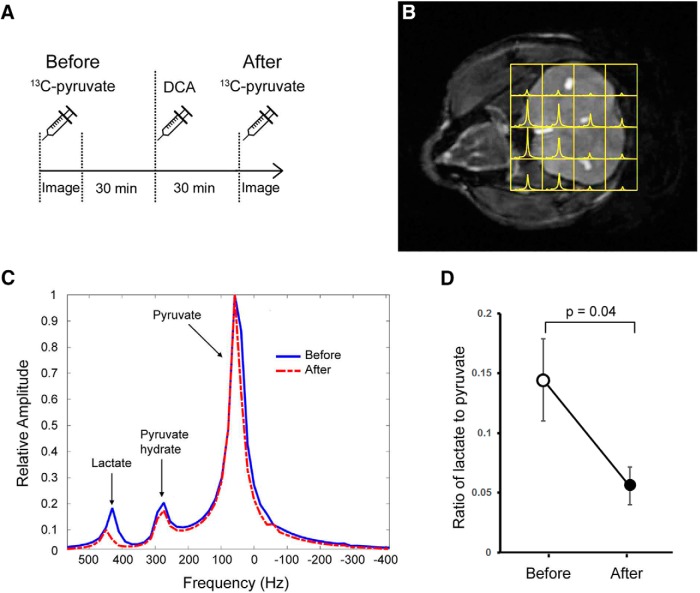
Hyperpolarized ^13^C-pyruvate magnetic resonance spectroscopic imaging reveals a decline in lactate following DCA administration. ***A***, Injection and imaging regime of 9-month-old mice using hyperpolarized MRSI of [1-^13^C] pyruvate to measure the conversion of pyruvate to lactate. Mice were imaged after the first ^13^C-pyruvate injection (Before) and 30 min later were then injected with DCA (200 mg/kg). Following a 30 min recovery time, another injection of ^13^C-pyruvate and imaging were performed (After). ***B***, ^1^H MRI of the brain in the coronal field overlayed with MRSI voxels containing spectra of ^13^C-labeled pyruvate and lactate (yellow). ***C***, Conversion of pyruvate to lactate was measured as a ratio of the observed lactate peak to pyruvate peak from before DCA injection (blue line) and after (red dashed line). A pyruvate hydrate peak was also recorded. ***D***, DCA injection reduces the ratio of lactate to pyruvate in the mouse brain (*p* = 0.04). Data shown are the mean ± SEM. *n* = 4.

To test whether DCA was a suitable compound for manipulating AG and lactate production in the brain, mice were analyzed using hyperpolarized ^13^C-pyruvate magnetic resonance spectroscopy 30 min before, and 30 min after, the injection of DCA (200 mg/kg) via an intravenous tail-vein catheter (Fig. 1*A*). ^13^C-pyruvate spectra were collected within individual voxels throughout the whole brain (Fig. 1*B*) and combined to calculate the ratio of lactate to pyruvate as an indication of pyruvate conversion to lactate in the mouse brain before and after DCA injection (Fig. 1*C*). The observed lactate-to-pyruvate metabolite ratio within the brain was significantly reduced from 0.144 ± 0.034 (mean ± SEM) before DCA exposure to 0.056 ± 0.016 after DCA administration (*t*_(1,10)_ = 2.753, *p* < 0.05; Fig. 1*D*). Thus, DCA administration reduces lactate production from pyruvate in the mouse brain 30 min after infusion, indicating that it is a suitable compound for inhibiting AG.

### DCA injection reduces the phosphorylation of PDH in the frontal cortex and hippocampus

**Figure 2.**
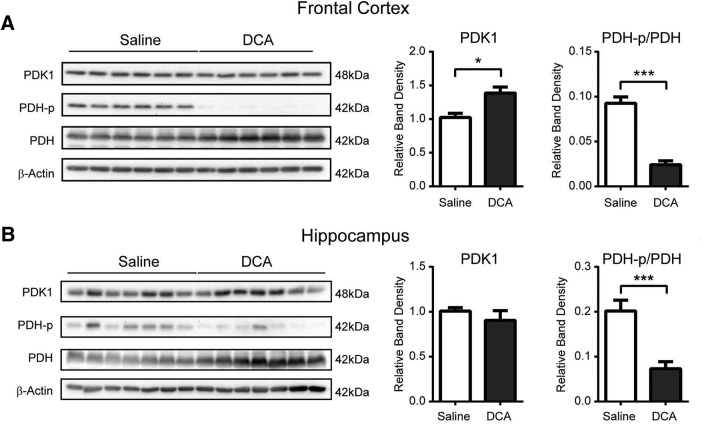
DCA injection reduces the phosphorylation of PDH in the frontal cortex and hippocampus. ***A***, ***B***, Western blot analysis (left) was performed on extracts from the frontal cortex (***A***) and the hippocampus (***B***) of mice intraperitoneally injected with either saline or DCA (200 mg/kg) 30 min before being killed. Densitometric analysis of Western blots (right) revealed significantly lower PDH phosphorylation in DCA-treated mice relative to saline-injected mice (*p < 0.05, ***p < 0.001; *n* = 6 and 6, respectively, for extracts from the frontal cortex of saline- and DCA-injected mice; and *n* = 7 and 7, respectively, for extracts from the hippocampus of saline- and DCA-injected mice). Band densities were standardized to β-actin controls. Data shown are the mean ± SEM.

To confirm that DCA exerts an effect on the brain regions related to spatial learning and memory, the phosphorylated form of PDH was measured in brain extracts from the frontal cortex and hippocampus 30 min after intraperitoneal injection of DCA. DCA administration caused a significant decrease in PDH phosphorylation levels in both the frontal cortex (*t*_(1,10)_ = 13.78, *p* < 0.001) and hippocampus (*t*_(1,14)_ = 4.493, *p* < 0.001) compared with saline-injected mice (Fig. 2). A significant decrease in PDK1 expression was also observed in the frontal cortex of DCA-injected mice (*t*_(1,10)_ = 2.837, *p* < 0.05). These results are in accordance with previous observations that DCA readily crosses the blood–brain barrier and targets AG by increasing PDH activity (Kato et al., 2008).

### DCA administration causes impairment in spatial learning

**Figure 3.**
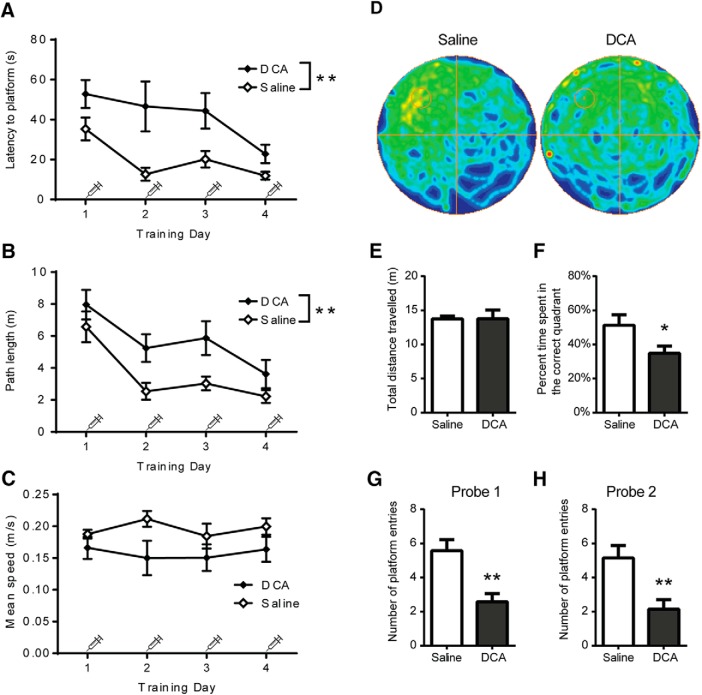
DCA administration causes impairment in spatial learning. Mice were intraperitoneally injected with saline or DCA (200 mg/kg) 30 min before each day of training and allowed to find the location of a hidden platform in the northwest quadrant. ***A–C***, The latency to find the platform (***A***), the total path length (***B***), and the mean speed (***C***) were recorded on each training day. ***D–G***, On day 5, a probe trial was performed without DCA injection, and mice were allowed to swim for 60 s. ***D***, The swim path for each group of mice was compiled into heat map representations. ***E–G***, Measurements were taken for the total distance traveled (***E***), the percentage of time spent in the correct quadrant (***F***), and the number of times the boundary of the platform was crossed (***G***). ***H***, A week after the first probe trial, mice were again tested with a second probe trial, and measurements were taken for the number of times the boundary of the platform was crossed. Data shown are the mean ± SEM. *p < 0.05, **p < 0.01. *n* = 7 and 7 for saline and DCA treatments, respectively.

To determine whether AG is required for spatial learning, a task that is dependent on communication between the hippocampus and frontal cortex, mice were injected intraperitoneally with DCA 30 min before each training session for the MWM task. Several measures were recorded for each training day, including the latency to find the platform, the total path length, and the mean speed (Fig. 3*A–C*). DCA administration resulted in a significant reduction in learning, as reflected by the increased latency time to find the platform (*F*_(1,12)_ = 9.83, *p* < 0.01) and increased path length (*F*_(1,12)_ = 12.84, *p* < 0.01), compared with saline-injected mice. There was no significant difference in mean speed between the two groups (*F*_(1,12)_ = 2.93, *p* = 0.11). Mice exposed to DCA during training subsequently exhibited a reduced ability to locate the platform during the probe trial, as visualized by a trace analysis of the swim path in a heat map (Fig. 3*D*). DCA-injected mice also displayed a significantly reduced percentage of time spent in the correct quadrant (*t*_(1,12)_ = 2.21, *p* < 0.05) and a reduction in the number of platform entries (*t*_(1,12)_ = 3.71, *p* < 0.05) compared with saline-injected mice, yet they displayed no difference in total distance covered during the trial (*t*_(1,12)_ = 0.01, *p* = 0.99; Fig. 3*E–G*). A second probe trial, performed 1 week later, revealed that DCA exposure during training impaired long-term memory, as indicated by a significant decrease in number of platform crosses (*t*_(1,12)_ = 3.25, *p* < 0.01) compared with saline-treated controls (Fig. 3*H*). Thus, DCA injection caused an impairment in learning that subsequently weakened memory association with the location of the platform both 24 h and 7 d after training.

### DCA administration does not interfere with memory recall

**Figure 4.**
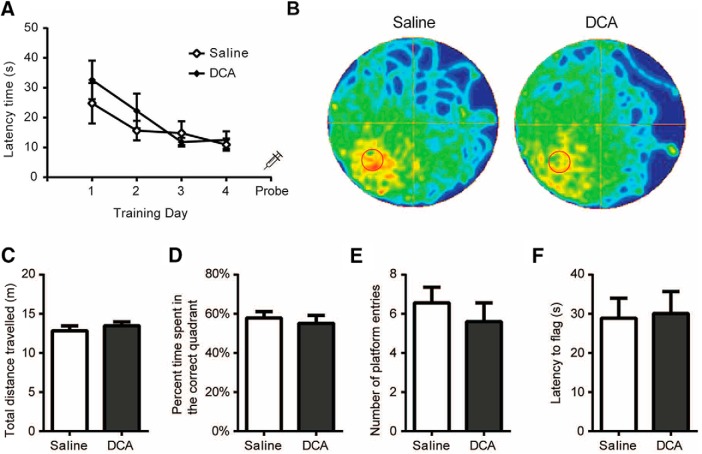
DCA administration does not interfere with memory retrieval. MWM performed by mice intraperitoneally injected with saline or DCA (200 mg/kg) on the probe trial. ***A***, Mice were trained for 4 consecutive days (4 trials/d) to find the location of a hidden platform in the southwest quadrant, and the latency to escape was recorded. ***B–G***, On day 5, a probe trial was performed in which the platform was removed and mice were allowed to swim for 60 s. ***B***, The swim path for each group of mice was recorded and compiled into heat map representations. ***C–E***, Measurements were taken for the total distance traveled (***C***), the percentage of time spent in the correct quadrant (***D***), and the number of times the boundary of the platform was crossed (***E***). ***F***, Immediately after the probe trial, a flag trial was performed and the latency to find the flag was recorded. Data shown are the mean ± SEM. *n* = 9 and 10 for saline and DCA treatments, respectively. No significant differences were observed between saline-treated (sham) and DCA-injected mice.

We next asked whether AG is required for spatial memory recall. Mice underwent 4 d of training in the MWM and were then injected intraperitoneally with DCA 30 min before the probe trial on the fifth day (Fig. 4*A*). DCA-injected mice showed no difference in memory performance from saline-injected mice, as demonstrated visually by a trace analysis of the swim path in a heat map (Fig. 4*B*). The total distance covered in the probe trial revealed no significant difference between saline- and DCA-injected mice (*t*_(1,16)_ = 0.80, *p* = 0.43; Fig. 4*C*). In addition, no significant differences in memory performance was observed, as indicated by the percentage of time spent in the correct quadrant (*t*_(1,16)_ = 0.52, *p* = 0.31) and the total number of platform entries (*t*_(1,16)_ = 0.77, *p* = 0.45; Fig. 4*D*,*E*). To confirm that DCA had no adverse effect on vision, a flag trial was performed that demonstrated that saline- and DCA-injected mice exhibited similar times to find the flag (*t*_(1,16)_ = 0.16, *p* = 0.44; Fig. 4*F*). These findings indicate that AG is not explicitly required for spatial memory retrieval. In addition, DCA injection had no adverse effects on swimming performance or visual acuity.

## Discussion

The role of glycogenolysis and lactate transport in learning and memory has been extensively studied (Magistretti and Allaman, 2018). Rats undergoing training during an inhibitory avoidance (IA) test exhibit a transient elevation of hippocampal lactate levels, measured by *in vivo* microdialysis, that is completely abolished by bilateral injection of the glycogenolysis inhibitor 1,4-dideoxy-1,4-imino-d-arabinitol (DAB) into the hippocampus (Suzuki et al., 2011). In a separate study, DAB injection in the rat hippocampus 5 min before behavioral testing significantly impaired spatial working memory in a four-arm spontaneous alternation task (Newman et al., 2017). Moreover, DAB-mediated impairment of spatial working memory was rescued by hippocampal injection of lactate (Newman et al., 2011). Chemical or genetic inhibition of monocarboxylate transporters that shuttle lactate in the brain also impairs IA and spatial working memory (Newman et al., 2011; Suzuki et al., 2011). Lactate derived from astrocytic glycogenolysis, and its subsequent transport to neurons, was shown to mediate hippocampal long-term potentiation (LTP) and to induce learning-dependent molecular changes, including increased expression of immediate early genes such as activity-regulated cytoskeletal protein (Arc), proto-oncogene c-Fos, early growth response 1 (Egr1), and phosphorylation of cAMP response element-binding protein and cofilin (Suzuki et al., 2011; Yang et al., 2014). Collectively, these observations provide strong support for the astrocyte neuron lactate shuttle model in which glucose, either directly or supplied via astrocytic glycogenolysis, is processed by AG within astrocytes to generate lactate, which is then transported to neurons to fuel the high energy demands associated with the cellular and molecular changes underlying memory formation and storage. However, the precise role of task-based activation of AG in memory acquisition and retrieval is poorly defined.

Several studies have demonstrated a task-associated decrease in extracellular glucose and a concurrent rise in extracellular lactate in the hippocampus during spatial memory processing (McNay et al., 2006, 2000; Newman et al., 2017). In addition, genes involved in AG, including LDHA and PDK, show increased hippocampal expression following IA learning, suggesting that the induction of enzymes that promote lactate production may be required for memory acquisition (Tadi et al., 2015). Rats also exhibit increased AG in the amygdala during IA training and memory testing (Sandusky et al., 2013). Interestingly, a marked increase in extracellular pyruvate was observed in the amygdala during IA training, whereas a significant drop in pyruvate was observed during memory testing (Sandusky et al., 2013). These observations prompted the authors to speculate that memory acquisition relies predominately on AG, whereas memory retrieval places a higher demand on oxidative metabolism. Our findings are in accordance with this theory, as discussed below.

In this study, we demonstrate for the first time that systemic DCA administration causes a decline in lactate production from pyruvate with a concomitant reduction in PDH phosphorylation in the frontal cortex and hippocampus, brain regions that are critical for spatial memory formation. In support of previous studies, we also show evidence that DCA can readily cross the BBB and inhibit PDK activity, thereby lowering PDH phosphorylation and reducing lactate production (Abemayor et al., 1984; Stacpoole et al., 2003). DCA injection before each training trial resulted in impaired learning, as reflected by the increased latency and increased total path length required to find the platform in the MWM. In addition, DCA-mediated inhibition of AG during training resulted in a reduced ability to locate the platform during subsequent probe trials. Although DCA exposure may potentially cause nonspecific effects (disorientation, and perceptual and motivational alterations), this is unlikely because we did not detect a significant difference in the mean speed during training or a decrease in the ability to find the flag during the probe trial following DCA injection. Interestingly, a single DCA injection before the probe trial had no effect on memory performance in mice that had already undergone regular training, indicating that AG is not explicitly required for memory retrieval. In light of these observations, we theorize that the transient increase in AG is required for the initial phases of learning, but not retrieval of established memories.

In support of this theory, several studies (Fox et al., 1988; Madsen et al., 1998, 1999) have shown that AG is quickly upregulated in response to neural stimulation and can account for up to 40% of the glucose consumed by active brain regions. In a recent study (Shannon et al., 2016), PET scans of humans before and after a visual–motor adaptation task revealed that the left Brodmann area 44 played a key role in task performance that correlated strongly with an increase in AG in the same brain region. Thus, elevated AG levels may arise to support the increased energetic demand in brain regions engaged in specific tasks. Moreover, AG plays a critical role in synaptic plasticity by enabling the synthesis of biosynthetic building blocks derived from glycolytic intermediates to support processes such as cytoskeletal rearrangements, lipid synthesis, protein trafficking, and increased gene expression (Yang et al., 2009; Bauernfeind et al., 2014; Bas-Orth et al., 2017). The spatial distribution of AG in the human brain also correlates with the expression of developmental and neotenous genes, strongly implicating AG in supporting new synapse formation and growth (Goyal et al., 2014). Indeed, recent studies (Yang et al., 2014; Margineanu et al., 2018) have shown that the exposure of mouse primary cortical neurons to lactate stimulates the expression of synaptic plasticity-related genes including *Arc*, *c-Fos*, *Bdnf*, and *Egr1* through an NMDA receptor-dependent mechanism.

Whether AG is upregulated directly in astrocytes, neurons, or both is currently a contentious issue (Dienel, 2012). Uptake of the glucose analog 6-deoxy-*N*-(7-nitrobenz-2-oxa-1,3-diazol-4-yl)-aminoglucose (NBDG), as assessed by *in vivo* two-photon imaging, revealed preferential uptake in astrocytes during activation of the somatosensory cortex in mice (Chuquet et al., 2010). Two-photon imaging of mouse hippocampal and cerebellar brain slices demonstrated that the transport and metabolism of the fluorescent glucose analog 2-NBDG is higher in astrocytes than in neurons located in their vicinity (Jakoby et al., 2014). In addition, confocal imaging of mouse primary cultures transfected with a genetically encoded FRET glucose biosensor revealed that glucose uptake is faster in astrocytes than neurons (Jakoby et al., 2014). However, several recent reports have provided evidence suggesting that glucose may also be taken up by neurons. Two-photon imaging analysis, using a near-infrared fluorophore, IRDye 800CW, conjugated to 2-deoxyglucose (2DG)-IR, revealed that 2DG-IR was taken up preferentially by neurons in awake behaving mice and that sensory stimulation leads to a pronounced increase in neuronal, but not astrocytic, 2DG-IR uptake (Lundgaard et al., 2015). However, it should be noted that 2DG-IR is likely internalized as a macromolecular complex with the glucose transporter Glu transporter (GluT) 1 by endocytosis and may not adequately reflect physiologic glucose uptake (Kovar et al., 2009). A separate study, using metabolic biosensors in hippocampal slices and brains of awake mice, revealed that neuronal stimulation is dependent on increased glucose consumption directly by neurons, not astrocytic-derived lactate (Díaz-García et al., 2017). Intrahippocampal injection of indinavir, a selective chemical inhibitor of the neuronal GluT4, before IA training in rats resulted in impaired learning, whereas inhibition of glucose transport after training had no effect on memory retrieval (Pearson-Leary and McNay, 2016). The discrepancies between astrocytic and neuronal uptake of glucose in the aforementioned studies may be attributed to a number of factors, including regional variation in glucose metabolism, the task-dependent nature of glucose uptake, and limitations when using glucose analogues and biosensors to measure glucose uptake and processing. Regardless, we propose that the transient elevation of neuronal glycolysis may act as a “first responder” to meet the increased energy needs of stimulated neurons due to the fact that glycolysis can generate ATP at a much faster rate than mitochondrial oxidative phosphorylation, while lactate production may spur the expression of immediate early genes involved in LTP.

Recent studies have shown that the glycolytic enzymes PDK1 and LDHA are primarily expressed within neurons of the frontal cortex and hippocampus of wild-type and transgenic Alzheimer’s disease mice (Harris et al., 2016; Zhang et al., 2018). Interestingly, it was recently shown that the induction of synaptic activity promotes increased neuronal glucose uptake and the expression of glycolytic genes (Bas-Orth et al., 2017; Segarra-Mondejar et al., 2018). Moreover, synaptic activity triggered increases in glucose metabolism and promotes the generation of lipid precursors required for cell membrane enlargement during neurite outgrowth (Segarra-Mondejar et al., 2018). It is possible that AG occurs in neurons during the initial phases of learning to promote structural synaptic remodeling within memory engram neurons. Once memory acquisition and synaptic remodeling have occurred, then mitochondrial oxidative phosphorylation may play a prominent role in ensuring that sufficient ATP production occurs to maintain established connections associated with memory recall. It is our intention that these findings will spur future studies to further dissect apart the role of either astrocytic or neuronal AG in various paradigms of learning and memory.

While our findings suggest that AG is required for memory acquisition, we could not determine the role of AG on memory consolidation due to the limitations of the experimental design used in this study. Memory consolidation has typically been evaluated using classical behavioral assays with rapid acquisition such as the passive-avoidance task (Lorenzini et al., 1996) or fear conditioning (Schafe et al., 1999). It is generally easier to differentiate post-training events, such as memory consolidation, from learning when the duration of the acquisition phase is short. The multiple training sessions used in the MWM over 4 d may result in memory traces that are reactivated and reconsolidated during each learning session, possibly obscuring events associated with the initial consolidation of a spatial memory (Florian and Roullet, 2004). However, several studies have made use of a massed training protocol for the MWM in which mice are submitted to four consecutive trial sessions within a 70–90 min period followed by a probe trial 24 h later (Florian and Roullet, 2004; Stein et al., 2014; Villain et al., 2016). The 1 d protocol can expose deficits in early consolidation after the training sessions without the complication of overlapping learning and consolidation processes during multiday training protocols (Stein et al., 2014). In future studies, it would be of interest to use the 1 d MWM training protocol and administer DCA after the condensed training sessions to determine whether the inhibition of AG also interferes with the early events associated with memory consolidation.

## Synthesis

Reviewing Editor: Jonathan Lee, University of Birmingham Decisions are customarily a result of the Reviewing Editor and the peer reviewers coming together and discussing their recommendations until a consensus is reached. When revisions are invited, a fact-based synthesis statement explaining their decision and outlining what is needed to prepare a revision will be listed below. The following reviewer(s) agreed to reveal their identity: Paul Gold, Pierre Magistretti.

The manuscript was recognised to address an important question concerning the contribution of aerobic glycolysis to phases of learning and memory, by using the administration of DCA to inhibit PDH activity. The data confirm a functional contribution to learning of spatial memory, but no necessity in the retrieval of a previously-learned spatial memory. The experiments were appropriately-designed to address the aims of the study, but the conclusions drawn either exceeded the scope of the experimental design or failed adequately to recognise and discuss potentially important caveats.

Major points:

1. The imaging component of the study was carried out in non-trained mice. The interpretative relevance of such a design to the effect of DCA on learning and memory is more removed than the alternative of measuring PDH phosphorylation post-learning or post-retrieval. This issue needs to be discussed carefully, as the lack of training imaging data constrains the interpretation of the DCA treatment results.

2. The design of the experiments in which DCA was administered prior to training trials does not allow any distinction between effects on learning and effects on consolidation. Without a post-training treatment condition, there is no way to link the effects of DCS to consolidation processes (especially in conjunction with the lack of training imaging data as already highlighted). Therefore, the discussion and abstract need to focus exclusively on addressing the effect of DCA on acquistion, and remove all reference to consolidation (other than perhaps linking to the literature supporting a role for aerobic glycolysis in consolidation in other memory settings). Similarly, the design and data do not allow any conclusions to be drawn in relation to reconsolidation.

3. The exclusive use of latency to the platform as the index of memory does not allow for a full exploration of the behavior and useful comparison to the literature. Additional measures (e.g. path length) should be included for openness and the benefit of the field.

4. The use of separate t-tests at each day is not the most appropriate statistical approach for the experimental design. We strongly recommend the use of factorial repeated-measures ANOVA for robustness.

Minor points:

1. It is noticeable to the attentive reader that the method of DCA adminstration differs across the experiments. This should be highlighted and briefly discussed.

2. An explicit discussion of whether the effects of DCA on task acquisition are genuinely due to learning (and not perceptual, motivational, disorientation, hyperthermia or other non-specific) effects would be very useful. The reviewers noted that the flag trial is useful for such a discussion.

3. Some methodological information is missing:

a. the intertrial interval during training

b. methods to keep the mice warm, e.g. toweling them, heat lamp, warm room temperature, etc.

c. On the probe trial, the mice start from the center. Where do they start on training trials?

4. There are a number of suggested corrections and clarifications to elements of the text and the representation of the literature:

Page 3, line 15 : “surprisingly” is not appropriate. The processes of memory consolidation and of memory recall are quite different and it is therefore likely, and not surprising, that they resort to different signals.

Page 7, line 5 : “lactate production from pyruvate” would be more appropriate as DCA does not directly influence the “conversion” of pyruvate to lactate.

Discussion line 10 : Newman et al., 2011, relates to spatial working memory.

Page 11 line 23 : Lundgaard et al 2015 : This study is marred by a major technical flaw. The labeled glucose used to visualize glucose “uptake” is actually internalized in neurons by endocytosis as a macromolecular complex with the glucose transporter, a phenomenon unrelated to dynamic glucose transport. (Kovar, J. L., Volcheck, W., Sevick-Muraca, E., Simpson, M. A. & Olive, D. M. Characterization and performance of a near-infrared 2-deoxyglucose optical imaging agent for mouse cancer models. Anal. Biochem. 384, 254-262 (2009).

This major technical issue should be mentioned if the authors wish to quote Lundgaard et al 2011.

Page 11 line 25 : no reference details for Bauernfeind et al., 2013. Furthermore the study focuses on primate brain and actually points to the importance of AG. Thus as stated in the text, this reference is incorrect.

Page 12 line 11 : very indirect evidence based on an indirect marker of metabolism, NAD/NADH. Furthermore the work of Suzuki et al and Newman et al clearly indicates the requirement of lactate transfer from astrocytes to neurons for memory consolidation.
